# Modelling the rheology of living cell cytoplasm: poroviscoelasticity and fluid-to-solid transition

**DOI:** 10.1007/s10237-024-01854-2

**Published:** 2024-07-08

**Authors:** Namshad Thekkethil, Jakub Köry, Ming Guo, Peter S. Stewart, Nicholas A. Hill, Xiaoyu Luo

**Affiliations:** 1 School of Mathematics and Statistics, University of Glasgow, Glasgow, UK; 2 Department of Mechanical Engineering, Massachusetts Institute of Technology, Cambridge, USA

**Keywords:** Cytoplasm, Rheology, Poroelasticity, Viscoelasticity

## Abstract

Eukaryotic cell rheology has important consequences for vital processes such as adhesion, migration, and differentiation. Experiments indicate that cell cytoplasm can exhibit both elastic and viscous characteristics in different regimes, while the transport of fluid (cytosol) through the cross-linked filamentous scaffold (cytoskeleton) is reminiscent of mass transfer by diffusion through a porous medium. To gain insights into this complex rheological behaviour, we construct a computational model for the cell cytoplasm as a poroviscoelastic material formulated on the principles of nonlinear continuum mechanics, where we model the cytoplasm as a porous viscoelastic scaffold with an embedded viscous fluid flowing between the pores to model the cytosol. Baseline simulations (neglecting the viscosity of the cytosol) indicate that the system exhibits seven different regimes across the parameter space spanned by the viscoelastic relaxation timescale of the cytoskeleton and the poroelastic diffusion timescale; these regimes agree qualitatively with experimental measurements. Furthermore, the theoretical model also allows us to elucidate the additional role of pore fluid viscosity, which enters the system as a distinct viscous timescale. We show that increasing this viscous timescale hinders the passage of the pore fluid (reducing the poroelastic diffusion) and makes the cytoplasm rheology increasingly incompressible, shifting the phase boundaries between the regimes.

## Introduction

Characterising cell rheology provides insight into their response to both internal and external stimuli, influencing important cellular processes such as migration, division, growth and differentiation (Alberts 2017; Ahmed and Betz 2015). Moreover, cell stiffness serves as a biomarker in diagnosing conditions like cancer (Maciaszek et al. 2011; Smelser et al. 2015; Grady et al. 2016). However, elucidating the rheology of living cells is challenging due to their complex and dynamic nature.

Eukaryotic cells comprise a stiff nucleus and cytoplasm enclosed within a plasma membrane. The cytoplasm includes both cytosol, a viscous fluid comprising a substantial portion of water, and a cytoskeleton (Alberts 2017; Clegg 1984) formed from cross-linked filamentous proteins including microtubules, actin filaments, and intermediate filaments (Brown and Tuszynski 1999). As a result, the rheological behaviour of cell cytoplasm is incredibly complex, characterized by a combination of mechanical responses including purely elastic (e.g. due to stretching of filaments, Gardel et al. 2004), viscoelastic (e.g. due to transient binding, unbinding and sliding of cross-links, Lieleg et al. 2009; Van Oosterwyck et al. 2013), compressible poroelastic (e.g. due to cytosol flow through the pores of the cytoskeleton, Keren et al. 2009) and viscous (e.g. due to viscosity of the cytosol, Mofrad and Kamm 2006; Vaziri and Gopinath 2008).

There have been a number of experimental techniques proposed to probe the rheological characteristics of the cell cytoplasm, including micropipette aspiration methods (Schmid-Schönbein et al. 1981), magnetic particle methods (Sato et al. 1984) and atomic force microscopy (Alcaraz et al. 2003). A recent study by one of us has explored the rheological response across a range of different timescales using optical tweezers (Hu et al. 2017), where the force required to move a bead through the cytoplasm is measured as a function of its displacement. To help in analysing these experiments, the present study constructs a comprehensive mathematical model for the motion of a bead through cell cytoplasm, where the rheological response is assumed to include elastic, viscoelastic, and viscous effects as well as local compressibility due to redistribution of fluid through the porous scaffold.

While the protein filaments which form the cytoskeleton are usually identified as the primary stress-resisting filaments (Satcher Jr and Dewey Jr 1996), simple elastic micro-filament networks are typically inadequate to describe the response to high-frequency deformations (Zaner 1995). As a result, most models of cell cytoplasm rheology focus primarily on the viscoelastic properties of the cytoskeleton. For example, viscoelastic models can be used to extract estimates of the stiffness (storage modulus) and viscosity (loss modulus) of the cytoskeletal network from experimental measurements (Zaner and Stossel 1982; Ziemann et al. 1994). In early theoretical models, these moduli were obtained using simple linear viscoelastic models (Schmid-Schönbein et al. 1981; Sato et al. 1984). However, nonlinearity becomes evident as the deformation amplitude increases, particularly at higher frequencies (Puig-de Morales-Marinkovic et al. 2007), while a power law relationship between stiffness and frequency (analogous to soft-glassy materials) has also been observed in some studies (Fabry et al. 2001; Puig-De-Morales et al. 2001; Trepat et al. 2004; Hecht et al. 2015). Notably, agreement between experiments and models has been increased by coupling nonlinear elastic and viscoelastic components together (Unterberger et al. 2013; Holzapfel 2002). We employ such a coupling in the model constructed below.

In more recent years, attention has also focussed on the dynamics of flow through the porous scaffold of the cytoskeleton, which has been shown to play a significant role in the mechanical response (Chandran and Barocas 2004) and possibly even dominate the rheological response when the pore size (set by the mesh spacing between filaments) is between 30 and 60 nm (Mitchison et al. 2008). Indeed, Hu et al. (2017) investigated cell behaviour across various timescales and found that while the viscoelastic response dominates at smaller strain rates, the dynamics of flow through the porous scaffold become dominant at larger strain rates. One possible approach to modelling such flows is to use poroelasticity theory (e.g. Zhang 2005; Kimpton et al. 2015; Copos and Guy 2018), which has been well validated against experiments (Moeendarbary et al. 2013). We incorporate poroelastic effects into the model constructed below.

The viscosity of the cytoplasm is typically attributed to the viscoelastic characteristics of the cytoskeleton discussed above, but the inherent viscosity of the cytosol may also play a significant role (Chee et al. 2008). This viscosity is typically assumed to be similar to that of water, but it should be noted that it can vary due to long-range ordering effects and the presence of hydrocarbon-rich zones (Keith and Snipes 1974), the presence of monomeric actin (Isenberg and Wohlfarth-Bottermann 1976), as well as the change in protein concentration linked to osmotic pressure differences (Li et al. 2020). Below, we use our theoretical model to study the influence of cytosol viscosity on cell rheology.

Despite a handful of experimental studies considering viscoelastic, poroelastic and viscous effects in cell cytoplasm rheology (Hu et al. 2017), a comprehensive modelling investigation combining all these mechanisms self-consistently is lacking (Köry et al. 2024). To this end, in Sect. 2, we propose a comprehensive mathematical model for the cytoplasm of living cells, incorporating nonlinear viscoelasticity within the cytoplasm coupled with a nonlinear poroelastic model for the motion of pore fluid; this model is then used to characterise the motion of an embedded circular bead to simulate optical tweezers experiments (Hu et al. 2017). In this model, nonlinear elasticity is incorporated using an upscaled continuum model derived rationally from discrete cytoskeletal modelling (Köry et al. 2024), while the poroelastic behaviour is modelled using the Brinkman–Darcy flow. In particular, we construct dimensionless groups which characterise the viscoelastic, poroelastic and viscous timescales relative to the prescribed motion of bead and conduct numerical simulations using a (computationally efficient) stabilized finite element method (FEM) (Thekkethil et al. 2023); we demonstrate that our model exhibits seven different rheological responses across the parameter space spanned by these relative timescales, in strong qualitative agreement with the experiments (Hu et al. 2017).

## Mathematical formulation

In our study, we represent the cytoplasm of a living cell as a cytoskeletal network comprising a cross-linked network of protein filaments (i.e. actin, intermediate filaments and microtubules) immersed in a viscous fluid medium (cytosol). In particular, the cytoplasm is modelled as a homogeneous poroviscoelastic medium, where the (viscous) pore fluid (i.e. cytosol) is contained within a viscoelastic matrix (i.e. cytoskeleton). We assume that the stress response of this matrix is anisotropic, with several embedded families of viscoelastic filaments mimicking the protein filaments, similar in spirit to the modelling of fibre-reinforced elastic tissues (Holzapfel and Ogden 2009). This mixture of fluid and matrix represents a single continuous poroviscoelastic medium, where the porosity $\phi$ is defined as the ratio between the volume of the fluid phase and the total volume.

For simplicity, in the following, we restrict attention to a planar region of the cell cytoplasm, well away from the nucleus and membrane. The material coordinates of the viscoelastic skeleton in the reference configuration are denoted as $\varvec{X}=(X,Y)$ and in the current configuration are represented by $\varvec{x}=(x,y)=\varvec{\chi }\left( \varvec{X},t\right)$ at time *t*. The deformation gradient tensor ${\textbf{F}}$ is computed with reference to the skeleton configuration

1 $$\begin{aligned} {\textbf{F}}=\frac{\partial \varvec{x}}{\partial \varvec{X}}. \end{aligned}$$

The volume occupied by the fluid $V_{\text{f}}(t)$ and the skeleton $V_{\text{s}}(t)$ at time *t* are given by

2 $$\begin{aligned} V_{\text{f}}(t) = \int _{\Omega ^t}\phi (\varvec{x},t)\text{d}\varvec{x}, \quad V_{\text{s}}(t) = \int _{\Omega ^t}(1-\phi (\varvec{x},t))\text{d}\varvec{x}, \end{aligned}$$

in which $\Omega ^t$ is the total volume occupied by the poroviscoelastic mixture in the current configuration. Hence, the volume of the pore fluid and the skeleton can also be computed with respect to the reference configuration as

3 $$\begin{aligned} V_{\text{f}}(t)= & {} \int _{\Omega ^0}J\phi \left( \varvec{\chi }\left( \varvec{X},t\right) ,t\right) \text{d}\varvec{X},\nonumber \\ V_{\text{s}}(t)= & {} \int _{\Omega ^0}J\left( 1-\phi \left( \varvec{\chi }\left( \varvec{X},t\right) ,t\right) \right) \text{d}\varvec{X}, \end{aligned}$$

in which $J=\text{det}\left( {\textbf{F}}\right)$ and $\Omega ^0$ is the volume occupied by the mixture in the reference configuration.

At a given point in the composite material $\varvec{x} \in \Omega ^t$, the local mass balance equations for the viscoelastic skeleton and the pore fluid are given as (Lee et al. 2016; Thekkethil et al. 2023)

4a $$\begin{aligned}&J\rho _{\text{s}}(1-\phi ) = \rho ^0_{\text{s}}\big (1-\phi ^0\big ), \end{aligned}$$

4b $$\begin{aligned}&J\rho _{\text{f}}\phi - \rho ^0_{\text{f}}\phi ^0 = m, \end{aligned}$$

in which $\rho _{\text{s}}$ and $\rho _{\text{f}}$ are the mass density of the viscoelastic skeleton and the pore fluid, respectively. Note that the superscript 0 denotes the reference configuration. Also, the added mass parameter *m* describes the change in the pore fluid mass with respect to the reference configuration per unit reference volume of the mixture. Given that the cytosol primarily comprises water (Clegg 1984), the fluid medium is assumed to be incompressible, with $\rho _{\text{f}}=\rho ^0_{\text{f}}$. Similarly, the viscoelastic skeleton is also assumed to be incompressible (Holzapfel et al. 2014; Wollrab et al. 2019), with $\rho _{\text{s}}=\rho ^0_{\text{s}}$. Thus, the local mass balance of the skeleton (Eq. 4) reduces to

5 $$\begin{aligned} J-1-\Delta V_{\text{f}}=0, \end{aligned}$$

in which $\Delta V_{\text{f}}=m/\rho ^0_{\text{f}}$ is the volume change ratio of the pore fluid with respect to the reference configuration.

The Eulerian form of the mass balance for the incompressible pore fluid is given as

6 $$\begin{aligned} \frac{\partial \phi }{\partial t}+\nabla \cdot \left( \phi \varvec{v}_{\text{f}}\right) = 0, \end{aligned}$$

where $\varvec{v}_{\text{f}}$ denotes the velocity of the pore fluid. Defining the perfusion velocity as the relative velocity between the pore fluid and the skeleton $\varvec{w}=\phi (\varvec{v}_{\text{f}}-\varvec{v}_{\text{s}})$, where $\varvec{v}_s$ is the velocity of the skeleton, the Lagrangian form of the mass balance for the pore fluid is obtained as (Thekkethil et al. 2023)

7 $$\begin{aligned} \frac{\text{d}\left( \Delta V_{\text{f}}\right) }{\text{d}t}+J\nabla \cdot \varvec{w}=0. \end{aligned}$$

To close the system, we link the perfusion velocity $\varvec{w}$ to the pressure in the fluid phase, denoted as pore pressure $p_{\text{pore}}$. In particular, we examine two different models for the pore fluid flow. Firstly, we examine Darcy flow in the pores (Darcy 1856), where

8 $$\begin{aligned} \varvec{w} = -k\nabla p_{\text{pore}}, \end{aligned}$$

in which *k* is the permeability of the porous medium. Note that in this poroelastic model, we assume that the fluid flow in the cytoplasm is solely driven by the pore pressure gradient due to the cytoplasmic deformation. This approximation is widely used in modelling porous tissues (Ricken et al. 2010; Richardson et al. 2021) and geomechanics (Wang 2000).

Secondly, we examine Darcy–Brinkman flow (Brinkman 1949), where

9 $$\begin{aligned} \varvec{w} = -k\nabla p_{\text{pore}}+ k\mu \nabla ^2\varvec{w}, \end{aligned}$$

in which $\mu$ is the uniform viscosity of the pore fluid. Note that in the case of Darcy flow, the viscous diffusion term is neglected, and there is free slip at the solid boundaries of the pores. Consequently, Darcy flow does not account for viscous drag between the pore fluid and the viscoelastic matrix.

Equations (7–9) are pulled back to the reference configuration of the skeleton to obtain the Lagrangian form of the mass and the momentum balance equations for the pore fluid, given as

10 $$\begin{aligned} \frac{\text{d}\left( \Delta V_{\text{f}}\right) }{\text{d}t} + \nabla _{\varvec{X}}\cdot \left( J{\textbf{F}}^{-1}\varvec{w}\right) =0, \end{aligned}$$

where for Darcy flow, we have

11 $$\begin{aligned} \varvec{w} =-k{\textbf{F}}^{-T}\nabla _{\varvec{X}}p_{\text{pore}}, \end{aligned}$$

while for Brinkman flow, we have

12 $$\begin{aligned} \varvec{w} =-k{\textbf{F}}^{-T}\nabla _{\varvec{X}}p_{\text{pore}}+\frac{k\mu }{J}\nabla _{\varvec{X}} \cdot \left( J{\textbf{C}}^{-1}\nabla _{\varvec{X}}\varvec{w}\right) , \end{aligned}$$

in which $\nabla _{\varvec{X}}$ represents the gradient with respect to the reference coordinate system $\varvec{X}=\left( X,Y\right)$.

For the poroviscoelastic mixture, neglecting the inertia and external body forces, the momentum balance equation in the Lagrangian frame is expressed in the form

13 $$\begin{aligned} \nabla _{\varvec{X}}\cdot \left( {\textbf{F}}{\textbf{S}}\right) =0, \end{aligned}$$

in which ${\textbf{S}}$ is the second Piola–Kirchhoff stress tensor.

To close the model, the stress tensor ${\textbf{S}}$ and the pore pressure $p_{\text{pore}}$ are obtained from the free energy function of the poroviscoelastic material, presented in the following section.

### Free energy density

We assume that the free energy density of the poroviscoelastic mixture, $\Psi$, can be expressed in a decoupled form (Coussy 1989; Lee et al. 2016), given as

14 $$\begin{aligned} \Psi = \Psi _{\text{fib}}+\Psi _{{\text{vol}}}+\Psi _{{\text{fl}}}, \end{aligned}$$

in which $\Psi _{\text{fib}}$ is the free energy of the viscoelastic skeleton, $\Psi _{{\text{vol}}}$ is a penalty term to enforce incompressibility of the material, and $\Psi _{{\text{fl}}}$ is the free energy of the pore fluid, which arises due to resistance to deformation of the skeleton by the pore fluid.

The free energy density of the viscoelastic skeleton, $\Psi _{\text{fib}}$, is determined by assuming an additive decomposition into viscoelastic and hyperelastic components. In particular, the free energy functional $\Psi _{\text{fib}}$ consists of a hyperelastic strain energy $\Psi _{{\text{hyp}}}$ and a configurational free energy of the viscoelastic part $\Psi _{\text{ve}}$ (Holzapfel 2002; Unterberger et al. 2013), given as

15 $$\begin{aligned} \Psi _{\text{fib}}\left( {\textbf{C}},\Gamma \right) = \Psi _{\text{hyp}}\left( \mathbf {\overline{C}}\right) +\Psi _{\text{ve}}\left( \mathbf {\overline{C}},\Gamma \right) , \end{aligned}$$

in which ${\textbf{C}}={\textbf{F}}^T{\textbf{F}}$ is the right Cauchy–Green strain tensor, and $\Gamma$ is a strain-like internal variable which characterises the viscoelastic relaxation (Holzapfel 2002). Note that the functions $\Psi _{\text{hyp}}$ and $\Psi _{\text{ve}}$ depend on the modified right Cauchy–Green strain tensor $\mathbf {\overline{C}}=J^{-2}{\textbf{F}}^T{\textbf{F}}$. Thus, $\Psi _{\text{hyp}}$ and $\Psi _{\text{ve}}$ represent the isochoric free energy densities of the hyperelastic and viscoelastic parts, respectively.

The hyperelastic strain energy density is derived as an upscaled continuum model of the cell cytoskeleton from a discrete network model for the constituent filaments (Köry et al. 2024). This model takes the form

16 $$\begin{aligned} \Psi _{\text{hyp}}\left( \mathbf {\overline{C}}\right) = \frac{c}{2} \left[ \left( \sqrt{\mathbf {f_{0}}\cdot \left( \mathbf {\overline{C}f_{0}}\right) } -\frac{1}{\alpha }\right) ^2+\left( \sqrt{\mathbf {s_{0}}\cdot \left( \mathbf {\overline{C}s_{0}}\right) }-\frac{1}{\alpha }\right) ^2 \right] , \end{aligned}$$

in which *c* is the filament stretching stiffness (defined in terms of the filament properties such as Young’s modulus, cross-sectional area and mesh spacing in Köry et al. (2024), with dimensions of stress), $\alpha$ is the initial pre-stretch of the filaments, and $\mathbf {f_{0}}$ and $\mathbf {s_{0}}$ are the initial filament directions in the reference configuration. In this case, we choose two families of filaments initially oriented with the *X* and *Y* axes, respectively. Köry et al. (2024) used a similar approach to model the cell cytoplasm during internal deformations, as a model for experiments probing cell rheology using optical tweezers (Hu et al. 2017), and showed that this regular arrangement did not change the outcome significantly. Köry et al. (2024) showed that with pre-stressed filaments, the force–displacement curve depends only weakly on the angle at which the bead is pulled through the network. They further showed that these force–displacement curves computed assuming square symmetry exhibit strong qualitative and quantitative agreement with force–displacement curves computed for disordered filament networks. Their observations suggest that our simplified initial filament arrangement is a reasonable approximation to real cytoskeletal networks. Furthermore, the pre-stretch parameter $\alpha$ arises from fixing the outer boundary to ensure that the total filament length exceeds its natural length. Despite the simplicity of this approach, the internal stress achieves self-equilibrium (Köry et al. 2024).

**Fig. 1:**
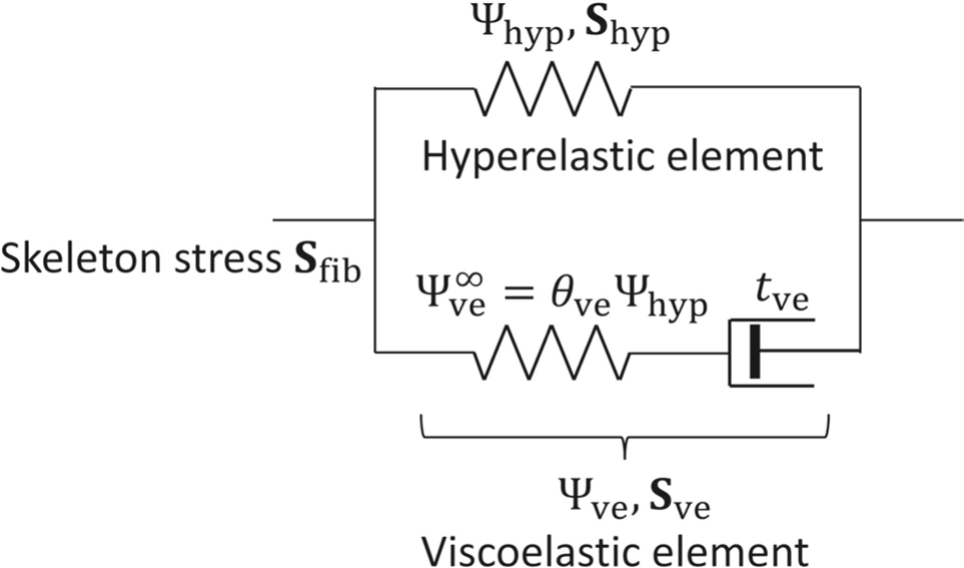
Rheological model of the viscoelastic filament

The viscoelastic component takes the form of a nonlinear one-element Maxwell model for viscoelasticity (Unterberger et al. 2013), as illustrated in Fig. 1. We assume that as time (denoted as *t*) approaches infinity, the limiting value of the configurational free energy $\Psi _{\text{ve}}$, denoted as $\Psi _{\text{ve}}^{\infty }$, corresponds to the purely hyperelastic response and can be expressed in terms of the hyperelastic strain energy function (Unterberger et al. 2013), in the form

17 $$\begin{aligned} \Psi _{\text{ve}}^{\infty }\left( \mathbf {\overline{C}}\right) =\theta _{\text{ve}}\Psi _{\text{hyp}}\left( \mathbf {\overline{C}}\right) , \end{aligned}$$

in which $\theta _{\text{ve}}$ is a non-dimensional parameter which depends on the viscoelastic relaxation time and measures the ratio of viscoelastic stress to hyperelastic stress (Unterberger et al. 2013).

For the incompressible poroviscoelastic mixture, the volumetric strain energy $\Psi _{\text{vol}}$, introduced as a penalty term using the hydrostatic pressure of the skeleton *p*, is a Lagrange multiplier written in terms of the change in volume of the skeleton in the form

18 $$\begin{aligned} \Psi _{\text{vol}}=-p\left( J-1-\Delta V_{\text{f}}\right) . \end{aligned}$$

The free energy of the pore fluid $\Psi _{\text{fl}}$ depends on the change in volume of the pores as the pore pressure changes. We derive the pressure–volume relationship for the pore fluid by assuming each pore is a spherical shell (see derivation in Appendix 1). The free energy density of the pore fluid $\Psi _{\text{fl}}$ is obtained as a function of the volume change ratio $\Delta V_{\text{f}}$, given as

19 $$\begin{aligned} \Psi _{\text{fl}}(\Delta V_{\text{f}})=\frac{2cH}{3R}\left[ \frac{3}{2}\left( 1+\Delta V_{\text{f}}\right) ^{2/3}+\frac{3}{4}\left( 1+\Delta V_{\text{f}}\right) ^{-4/3}\right] . \end{aligned}$$

Hence, the total free energy density of the poroviscoelastic medium is given as

20 $$\begin{aligned} \Psi \left( {\textbf{C}},\Gamma ,\Delta V_{\text{f}}\right) =\Psi _{\text{hyp}}\left( \mathbf {\overline{C}}\right) +\Psi _{\text{ve}}\left( \mathbf {\overline{C}}, \Gamma \right) +\Psi _{\text{vol}}\left( J,\Delta V_{\text{f}}\right) +\Psi _{\text{fl}}\left( \Delta V_{\text{f}}\right) . \end{aligned}$$

From the above expression, the total pore pressure $p_{\text{pore}}$ of the poroviscoelastic medium is obtained as

21 $$\begin{aligned} p_{\text{pore}}=\frac{\partial \Psi }{\partial \Delta V_{\text{f}}}=p+p_{\text{fl}}. \end{aligned}$$

The corresponding second Piola–Kirchhoff stress tensor, ${\textbf{S}}$, is obtained as

22 $$\begin{aligned} {\textbf{S}}= & {} 2\frac{\partial \Psi }{\partial {\textbf{C}}}={\textbf{S}}_{\text{hyp}}-pJ{\textbf{C}}^{-1}+{\textbf{S}}_{\text{ve}}, \text{where }{\textbf{S}}_{\text{hyp}}=2\frac{\partial \Psi _{\text{hyp}}}{\partial {\textbf{C}}},\nonumber \\ {\textbf{S}}_{\text{ve}}= & {} 2\frac{\partial \Psi _{\text{ve}}}{\partial {\textbf{C}}}. \end{aligned}$$

The viscoelastic stress ${\textbf{S}}_{\text{ve}}$ is obtained from the rheology model illustrated in Fig. 1. Using the assumption given in Eq. (17), the second Piola–Kirchhoff stress of the elastic component of the Maxwell element (Fig. 1) is given as ${\textbf{S}}_{\text{ve}}^{\infty }=\theta _{\text{ve}}{\textbf{S}}_{\text{hyp}}$. Thus, the evolution equation for the viscoelastic stress ${\textbf{S}}_{\text{ve}}$ is given as (Holzapfel 2002)

23 $$\begin{aligned} \frac{\text{d}{\textbf{S}}_{\text{ve}}}{\text{d}t}+\frac{{\textbf{S}}_{\text{ve}}}{t_{\text{ve}}} =\theta _{\text{ve}}\frac{\text{d}{\textbf{S}}_{{\text{hyp}}}}{\text{d}t}, \end{aligned}$$

in which $t_{\text{ve}}$ is the viscoelastic relaxation time of the skeleton. The above equation is solved to obtain the viscoelastic stress at a time instant $t=T$, given as

24 $$\begin{aligned} {\textbf{S}}_{\text{ve}}&= {\textbf{S}}_{\text{ve},0}\exp {\left( -T/t_{\text{ve}}\right) }\nonumber \\ & \quad + \theta _{\text{ve}}\int _0^T\exp {\left[ -(T-t)/t_{\text{ve}}\right] }\frac{\text{d}{\textbf{S}}_{\text{hyp}}}{\text{d}t}\text{d}t \end{aligned}$$

in which ${\textbf{S}}_{\text{ve},0}$ is the viscoelastic stress at time $t=0$. Note that the viscoelastic effects depend on the path and the loading history, as indicated by the integral over time.

In summary, the dependent variables of the model are the displacement vector $\varvec{u}=\varvec{x}-\varvec{X}$, the hydrostatic pressure *p*, the volume change ratio of the pore fluid $\Delta V_{\text{f}}$, the total pore pressure $p_{\text{pore}}$, and the Darcy velocity $\varvec{w}$. The corresponding governing equations are

25 $$\begin{aligned}{} & {} \nabla _{\varvec{X}}\cdot \left[ {\textbf{F}}\left( {\textbf{S}}_{{\text{hyp}}}+{\textbf{S}}_{\text{ve}}\right) \right] -\nabla _{\varvec{X}}\cdot \left( pJ{\textbf{F}}^{-T}\right) =0, \end{aligned}$$

26 $$\begin{aligned}{} & {} J-1-\Delta V_{\text{f}}=0, \end{aligned}$$

27 $$\begin{aligned}{} & {} \frac{\text{d}\left( \Delta V_{\text{f}}\right) }{\text{d}t}+\nabla _{\varvec{X}}\cdot \left( J{\textbf{F}}^{-1}\varvec{w}\right) =0, \end{aligned}$$

28 $$\begin{aligned}{} & {} p_{\text{pore}}=p+\frac{2cH}{3R}\left[ (1+\Delta V_{\text{f}})^{-1/3}-(1+\Delta V_{\text{f}})^{-7/3}\right] , \end{aligned}$$

29 $$ \varvec{w} = {\left\{  \begin{aligned} &  -k{\textbf{F}}^{-T}\nabla _{\varvec{X}}p_{\text{pore}} \qquad \qquad \qquad \qquad\quad\quad\quad\ \text{for Darcy Flow}, \\& -k{\textbf{F}}^{-T}\nabla _{\varvec{X}}p_{\text{pore}}\\& \quad +k\mu J^{-1}\nabla _{\varvec{X}}\cdot \left[ \nabla _{\varvec{X}}\left( J{\textbf{C}}^{-1}\varvec{w}\right) \right]  \qquad \qquad \text{for Brinkman flow}.
\end{aligned}  \right. }$$

The non-dimensional governing equations and the corresponding non-dimensional parameters for the model of the cytoplasm are presented in the following section.

### Non-dimensionalization

In the simulations below, we choose the side length of this square large enough so that the boundaries do not influence the results. Computational experiments are carried out by moving a circular bead of diameter *a* inside the model cell at a constant velocity *V* to mimic the optical tweezers experiments for characterising rheology (Hu et al. 2017).

We scale all length scales on *a*, velocities on *V*, time on the experimental timescale *a*/*V*, and stresses and pressures on the filament stretching stiffness *c*. The non-dimensional form of the governing equations (Eqs. 25–29) is given by

30 $$\begin{aligned}{} & {} \nabla ^*_{\varvec{X}}\cdot \left[ {\textbf{F}}\left( {\textbf{S}}_{{\text{hyp}}}^{*}+{\textbf{S}}_{\text{ve}}^{*}\right) \right] -\nabla ^*_{\varvec{X}}\cdot \left( p^*J{\textbf{F}}^{-T}\right) =0, \end{aligned}$$

31 $$\begin{aligned}{} & {} J-1-\Delta V_{\text{f}}=0, \end{aligned}$$

32 $$\begin{aligned}{} & {} \frac{\text{d}\left( \Delta V_{\text{f}}\right) }{\text{d}t^*}+\nabla _{\varvec{X}}^*\cdot \left( J{\textbf{F}}^{-1}\varvec{w}^*\right) =0, \end{aligned}$$

33 $$\begin{aligned}{} & {} p_{\text{pore}}^*=p^*+\frac{2H}{3R}\left[ (1+\Delta V_{\text{f}})^{-1/3}-(1+\Delta V_{\text{f}})^{-7/3}\right] , \end{aligned}$$

34 $$\varvec{w}^* = {\left\{ \begin{aligned} & -\frac{1}{\tau _{\text{pe}}}{\textbf{F}}^{-T}\nabla _{\varvec{X}}^*p_{\text{pore}}^* \quad\quad\quad\quad\quad\quad\quad\quad  \text{for Darcy Flow}, \\&  \frac{1}{\tau _{\text{pe}}}\left( -{\textbf{F}}^{-T}\nabla _{\varvec{X}}^*p_{\text{pore}}^*+\right. \\& \left. \quad \frac{\tau _{\text{vis}}}{J}\nabla ^*_{\varvec{X}}\cdot \left[ \nabla ^*_{\varvec{X}}\left( J{\textbf{C}}^{-1}\varvec{w}^*\right) \right] \right) \quad \quad\quad \text{for Brinkman flow}, \end{aligned}\right. }$$

in which the superscript $^*$ represents the non-dimensional version of the same quantity. The non-dimensional form of the viscoelastic stress ${\textbf{S}}_{\text{ve}}^{*}$ at time $T^*$ is given as

35 $$\begin{aligned} {\textbf{S}}_{\text{ve}}^*&= {\textbf{S}}_{\text{ve},0}^*\exp {\left( -T^*/\tau _{\text{ve}}\right) }\nonumber \\ &\quad + \theta _{\text{ve}}\int _0^{T^*}\exp {\left[ -(T^*-t^*)/\tau _{\text{ve}}\right] } \frac{\text{d}{\textbf{S}}_{\text{hyp}}^*}{\text{d}t^*}\text{d}t^* \end{aligned}$$

The non-dimensional governing parameters are summarised in Table 1. In the Darcy flow model, there are two significant non-dimensional timescales to consider: the non-dimensional poroelastic timescale $\tau _{\text{pe}}$, and the non-dimensional viscoelastic timescale $\tau _{\text{ve}}$. These are similar to the two timescales considered in the experimental study of Hu et al. (2017). Here, $\tau _{\text{pe}}$ represents the ratio of the poroelastic diffusion timescale $a^2/(ck)$ of the cytoplasm [approximately 0.02–0.1 s, Hu et al. (2017)] to the experimental timescale *a*/*V*, while $\tau _{\text{ve}}$ denotes the ratio of the viscoelastic relaxation time of the cytoskeleton, $t_{\text{ve}}$ [of the order of 1.0 s, Hu et al. (2017)], to the experimental timescale. In our study with the Brinkman flow model, we also consider a new third non-dimensional timescale, the viscous timescale $\tau _{\text{vis}}$, arising due to the viscous effects of the cytosol (pore fluid) that represents the ratio of the viscous diffusion timescale $\mu /c$ to the experimental timescale *a*/*V*.

We select the parameters for our model based on insights from various experimental and modelling studies found in the literature. Following the approach of Hu et al. (2017), we investigate the influence of non-dimensional poroelastic and viscoelastic timescales, $\tau _{\text{pe}}$ and $\tau _{\text{ve}}$, across a range of plausible values. We maintain the non-dimensional parameter $\theta _{\text{ve}}$ at a constant value of 0.835, consistent with values determined by Unterberger et al. (2013) determined through experimental data on a viscoelastic actin network. Similarly, we set the pre-stretch parameter $\alpha$ to a constant value of 1.012 for most simulations, aligning with the findings of Unterberger et al. (2013). We also explore the impact of varying this pre-stretch in Appendix 2. Furthermore, since water constitutes approximately 80% of the cytoplasm (Shepherd 2006) and the estimated pore dimension of the cytoskeletal network is approximately 50 nm (Luby-Phelps 1999), we set the initial porosity to $\phi =0.8$ and the pore thickness to radius ratio $H/R=0.077$.

In simulations, we set the filament stretching stiffness, *c*, to $10^3$ Pa (Satcher Jr and Dewey Jr 1996). Additionally, we assume the cytosol viscosity $\mu$ to be similar to that of water ($\mu \approx 10^{-3}$ Pa s, Clegg 1984). Consequently, the viscous diffusion timescale is roughly $10^{-6}$ s, notably shorter than both the poroelastic and viscoelastic timescales. Furthermore, in Sect. 5.2, we analyse the impact of varying viscous timescales since the viscosity can be influenced by a number of factors (Keith and Snipes 1974; Li et al. 2020).

**Table 1:** Non-dimensional governing parameters for the mathematical model of the cytoplasm

Non-dimensional parameter	Expression	Value
Poroelastic timescale ratio	$\tau _{\text{pe}}=\frac{Va}{ck}$	$10^{-3} - 2\times 10^2$
Viscoelastic timescale ratio	$\tau _{\text{ve}}=\frac{Vt_{\text{ve}}}{a}$	$10^{-4} - 5\times 10^3$
Viscous timescale ratio	$\tau _{\text{vis}}=\frac{V\mu }{ca}$	$10^{-6} - 10^{-4}$
Viscoelastic to elastic stress ratio	$\theta _{\text{ve}}$	0.835
Pre-stretch parameter	$\alpha$	1–1.1
Initial porosity	$\phi _0$	0.8
Pore thickness to radius ratio	*H*/*R*	0.125

## Numerical formulation

The non-dimensional governing equations (Eqs. 30–34) are solved using a Galerkin finite element method. The corresponding weak formulation of the governing equations takes the form

36a $$\begin{aligned} 0&= \int _{\Omega ^0} \left[ {\textbf{F}}\left( {\textbf{S}}_{\text{hyp}}^*+{\textbf{S}}^*_{\text{ve}}\right) -p^*J{\textbf{F}}^{-T}\right] \cdot \nabla _{\varvec{X}}^{*}\tilde{\varvec{u}} \text{ d}\varvec{X}\nonumber \\&\quad -\int _{\Gamma ^0}\varvec{t}_0\cdot \tilde{\varvec{u}}\text{ d}A, \end{aligned}$$

36b $$\begin{aligned} 0&= \int _{\Omega ^0}\left( J-1-\Delta V_{\text{f}}\right) {\tilde{p}} \text{ d}\varvec{X}, \end{aligned}$$

36c $$\begin{aligned} 0&= \int _{\Omega ^0}\left[ \frac{\text{d}\left( \Delta V_{\text{f}}\right) }{\text{d}t^*}\tilde{\nu }-\left( J{\textbf{F}}^{-1}\varvec{w}^*\right) \cdot \nabla \tilde{\nu }\right] \text{ d}\varvec{X}\nonumber \\&\quad +\int _{\Gamma _0}\left( J{\textbf{F}}^{-1}\varvec{w}^*\cdot \varvec{n}\right) \cdot \tilde{\nu }\text{ d}A, \end{aligned}$$

36d $$\begin{aligned} 0&= \int _{\Omega ^0}\left[ p_{\text{pore}}^*-p^*-\frac{2H}{3R}\left[ (1+\Delta V_{\text{f}})^{-1/3}-(1+\Delta V_{\text{f}})^{-7/3}\right] \right] \nonumber \\&\quad \tilde{p_{\text{pore}}}\text{ d}\varvec{X}, \end{aligned}$$

36e $$\begin{aligned} 0&= \int _{\Omega ^0}\left( \varvec{w}^*+\frac{{\textbf{F}}^{-T}}{\tau _{\text{pe}}}\nabla _{\varvec{X}}^* p_{\text{pore}}^*\right) \cdot \tilde{\varvec{w}}\text{ d}\varvec{X}, \end{aligned}$$

36f $$\begin{aligned} 0&= \int _{\Omega ^0}\left( \varvec{w}^*+\frac{{\textbf{F}}^{-T}}{\tau _{\text{pe}}}\nabla _{\varvec{X}}^*p_{\text{pore}}^*\right) \cdot \tilde{\varvec{w}}\text{ d}\varvec{X}\nonumber \\&\quad +\int _{\Omega ^0}\frac{\tau _{\text{vis}}}{J\tau _{\text{pe}}}\nabla _{\varvec{X}}^* \left( J{\textbf{C}}^{-1}\varvec{w}^*\right) \cdot \nabla _{\varvec{X}}^*\tilde{\varvec{w}}\text{ d}\varvec{X} \nonumber \\&\quad -\int _{\Gamma ^0}\left[ \frac{\tau _{\text{vis}}}{J\tau _{\text{pe}}}\nabla _{\varvec{X}}^* \left( J{\textbf{C}}^{-1}\varvec{w}^*\right) \cdot \varvec{n}\right] \cdot \tilde{\varvec{w}}\text{ d}A, \nonumber \end{aligned}$$

in which $\Gamma ^0$ is the boundary of the domain $\Omega ^0$ in the reference configuration. The test functions $\tilde{\varvec{u}}$, ${\tilde{p}}$, $\tilde{\nu }$, $\tilde{p_{\text{pore}}}$, and $\tilde{\varvec{w}}$ correspond to the non-dimensional displacement vector $\varvec{u}^*$, pressure $p^*$, volume change ratio $\Delta V_{\text{f}}$, pore pressure $p_{\text{pore}}^*$, and Darcy velocity vector $\varvec{w}^*$, respectively.

We note that, due to the incompressibility condition (Eq. 36b), the full system (Eq. 36) does not satisfy the Ladyzhenskaya–Babuska–Brezzi (LBB) criterion (Brezzi and Fortin 2012), resulting in numerical instability. To stabilise the formulation, we implement an efficient stabilized finite element method using piece-wise linear ($P^1$) finite elements (Klaas et al. 1999; Rossi et al. 2016; Thekkethil et al. 2023). Specifically, we adopt a stabilization method developed for $P^1$ elements spanning an isotropic hyperelastic material (Klaas et al. 1999), and here apply it to an anisotropic poroviscoelastic material. In this method, the residual of the mass balance (Eq. 36b) is modified using a perturbation term with a small-scale component of displacement $\varvec{u}'$, given as

37 $$\begin{aligned} 0&=\int _{\Omega ^0}\left( J-1-\Delta V_{\text{f}}\right) {\tilde{p}} \text{ d}\varvec{X}+\sum _{K\in {\mathcal {T}}^h}\int _K\left( \varvec{u}'\cdot {\textbf{H}}\nabla _{\varvec{X}}^*{\tilde{p}}\right) \text{ d}\varvec{X}, \nonumber \\ \text{where}\quad \varvec{u}'&=-\frac{\alpha _s {h_e^*}^2c}{2\mu _e}{\textbf{F}}^{-T}\nabla _{\varvec{X}}^*p^*, \end{aligned}$$

in which *K* is an element of the triangulation ${\mathcal {T}}^h$, such that ${\bar{\Omega }} = \cup _{K\in {\mathcal {T}}^h}K$ (Rossi et al. 2016). For the two-dimensional model considered here, *K* represents triangular elements. Furthermore, $\alpha _s$ is a non-dimensional non-negative stability parameter depending on the element type (Klaas et al. 1999), $h_e^*$ is the characteristic non-dimensional length of the element, and $\mu _e$ is the shear modulus of the viscoelastic skeleton, obtained as (Scovazzi et al. 2016)

38 $$\begin{aligned} \mu _e=\frac{\partial \Psi _{\text{hyp}}}{\partial \overline{I}_{\text{4f}}}+\frac{\partial \Psi _{\text{hyp}}}{\partial \overline{I}_{\text{4s}}} \end{aligned}$$

in which $\overline{I}_{\text{4f}}=\mathbf {f_{0}}\cdot \left( \mathbf {\overline{C}f_{0}}\right)$, and $\overline{I}_{\text{4s}}=\mathbf {s_{0}}\cdot \left( \mathbf {\overline{C}s_{0}}\right)$. Through numerical experiments, we determined that the method is stabilized without excessive dissipative effects strongly influencing the outcome by setting $0.1\le \alpha _s\le 0.3$.

The stabilized weak form of the governing equations (Eqs. 36a, 37, 36c–36f) are solved simultaneously using a fully implicit method. To advance from a time instant *n* to $n+1$ with a constant time step $\Delta t^*$, the second-order midpoint method is used for time integration. The resulting nonlinear equations are solved using Newton’s method.

The total second Piola Kirchhoff stress a the $(n+1)^\text{th}$ time step is given by

39 $$\begin{aligned} {\textbf{S}}^{*,n+1}={\textbf{S}}_{{\text{hyp}}}^{*,n+1}+{\textbf{S}}_{{\text{vol}}}^{*,n+1}+{\textbf{S}}_{\text{ve}}^{*,n+1}. \end{aligned}$$

Equation (35) is integrated from $t=0$ to $t=t^{n+1}$ to obtain the non-dimensional viscoelastic stress ${\textbf{S}}_{\text{ve}}^{*,n+1}$ in the form

40 $$\begin{aligned} {\textbf{S}}_{\text{ve}}^{*,n+1}= & {} \exp \left( -\frac{\Delta t^*}{\tau _{\text{ve}}}\right) {\textbf{S}}_{\text{ve}}^{*,n}+ \\{} & \exp \left( -\frac{\Delta t^*}{2\tau _{\text{ve}}}\right) \theta _{\text{ve}} \left( {\textbf{S}}_{{\text{hyp}}}^{*,n+1} -{\textbf{S}}_{{\text{hyp}}}^{*,n}\right) . \end{aligned}$$

As is typical for strongly nonlinear problems, the choice of time step size becomes crucial for ensuring the convergence of the Newton solver. To ensure the convergence, an empirical band for the non-dimensional time step $\Delta t^*$ has been estimated as follows:

41 $$\begin{aligned} \Delta t^* = \min \left( 0.01\tau _{\text{ve}},\alpha _{\text{CFL}}\underset{e\in {\mathcal {T}}^{h}}{\min }h_{e}^*\right) . \end{aligned}$$

Here, the first term on the right-hand side ensures that the timescale of the bead movement, *a*/*V*, is significantly shorter than the viscoelastic timescale. The second term guarantees that the bead’s displacement within a single time step does not exceed one grid space. This is analogous to the Courant–Friedrichs–Lewy (CFL) condition, characterized by the CFL number $\alpha _{\text{CFL}}$. In our numerical simulations, where we use $0.1\le \alpha _{\text{CFL}}\le 1.0$, the nonlinear solver generally converges to acceptable tolerance within five Newton iterations.

## Computational model

**Fig. 2:**
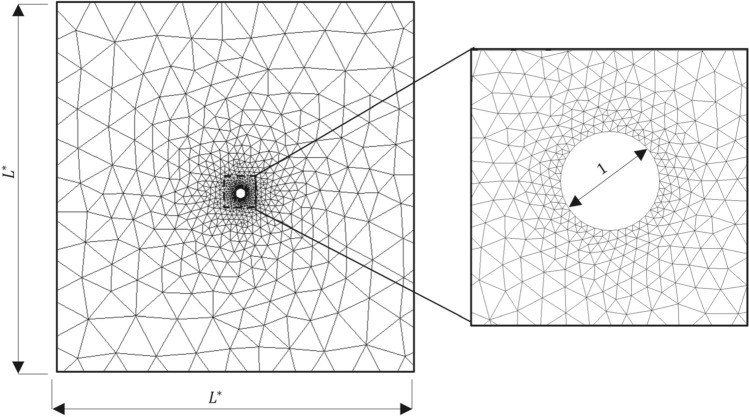
Non-dimensional computational domain for the movement of a bead inside the cytoplasm

We conduct numerical simulations for a standard stress relaxation test like the optical tweezers experiments conducted by Hu et al. (2017). This test involves initially moving a bead within the cytoplasm at a constant velocity, followed by holding the bead stationary to allow the cytoplasm to relax. Figure 2 shows the non-dimensional computational domain for the stress-relaxation test in which the numerical simulations are conducted using our stabilised FEM. In accordance with a typical stress relaxation test (Hu et al. 2017), the bead is first moved parallel to the *X*-axis by a fixed distance (which we chose as $\Delta x^*=0.1$) and then held stationary.

A prescribed displacement boundary condition is used at the bead surface, whereas a traction-free boundary condition is used at the other boundaries. A no-penetration condition is used on all the boundaries for the pore fluid flow. An additional no-slip boundary condition is also used on all the surfaces for the Brinkman flow.

Following the experimental studies using a stress relaxation test, we primarily characterise the rheological properties of our model cytoplasm by analysing the total force acting on the bead. The total non-dimensional force acting on the bead surface $\varvec{F}^*$ is computed as the sum of the force per unit area acting on the skeleton $\varvec{F}_{\text{sk}}^*$ and the pore fluid $\varvec{F}_{\text{pore}}^*$. The skeleton force per unit area $\varvec{F}_{\text{sk}}^*$ is given as

42 $$\begin{aligned} \varvec{F}_{\text{sk}}^* = \frac{1}{\pi }\int _{\Gamma _{\text{b}}}\left( 1-\phi _{\text{b}}\right) \varvec{\sigma }_{\text{b}}^*\cdot \varvec{\hat{n}}{\textrm{d}}\Gamma _{\text{b}}, \end{aligned}$$

in which the subscript ${\text{b}}$ represents the bead surface, $\varvec{\sigma }_{\text{b}}^*=J^{-1}{\textbf{F}}{\textbf{S}}_{\text{b}}^*{\textbf{F}}^T$ is the total Cauchy stress, and $\varvec{\hat{n}}$ corresponds to the outward pointing unit vector normal to the surface of the bead. The total pore fluid force per unit area $\varvec{F}_{\text{pore}}^*$ consists of two components: the force due to the pore pressure, $\varvec{F}_{\text{pr}}^*$, and the viscous drag force, $\varvec{F}_{\text{vis}}^*$, given as

43 $$\begin{aligned} \varvec{F}_{\text{pore}}^* & = \varvec{F}_{\text{pr}}^*+\varvec{F}_{\text{vis}}^* \nonumber \\ \text{where}\quad \varvec{F}_{\text{pr}}^* &= -\frac{1}{\pi }\int _{\Gamma _{\text{b}}}\phi _{\text{b}} p_{\text{pore,b}}^* \varvec{\hat{n}}{\textrm{d}}\Gamma _{\text{b}}, \nonumber \\ \text{and}\quad \varvec{F}_{\text{vis}}^* &={\left\{ \begin{array}{ll} \varvec{0} &{} \text{for Darcy flow},\\ \frac{1}{\pi }\int _{\Gamma _{\text{b}}}\phi _{\text{b}} \varvec{\sigma }_{\text{vis,b}}^* \cdot \varvec{\hat{n}} {\textrm{d}}\Gamma _{\text{b}} &{} \text{for Brinkman flow}, \end{array}\right. } \end{aligned}$$

in which $\varvec{\sigma }_{\text{vis,b}}^*$ is the viscous stress on the bead surface evaluated as

44 $$\begin{aligned} \varvec{\sigma }_{\text{vis,b}}^*=\tau _{\text{vis}} \nabla _{\varvec{X}}^*\varvec{w}^*_{\text{b}}. \end{aligned}$$

It should be noted that the presence of the viscous stress term is specific to the Brinkman flow model. In contrast, the Darcy flow model neglects the additional viscous stress.

**Fig. 3:**
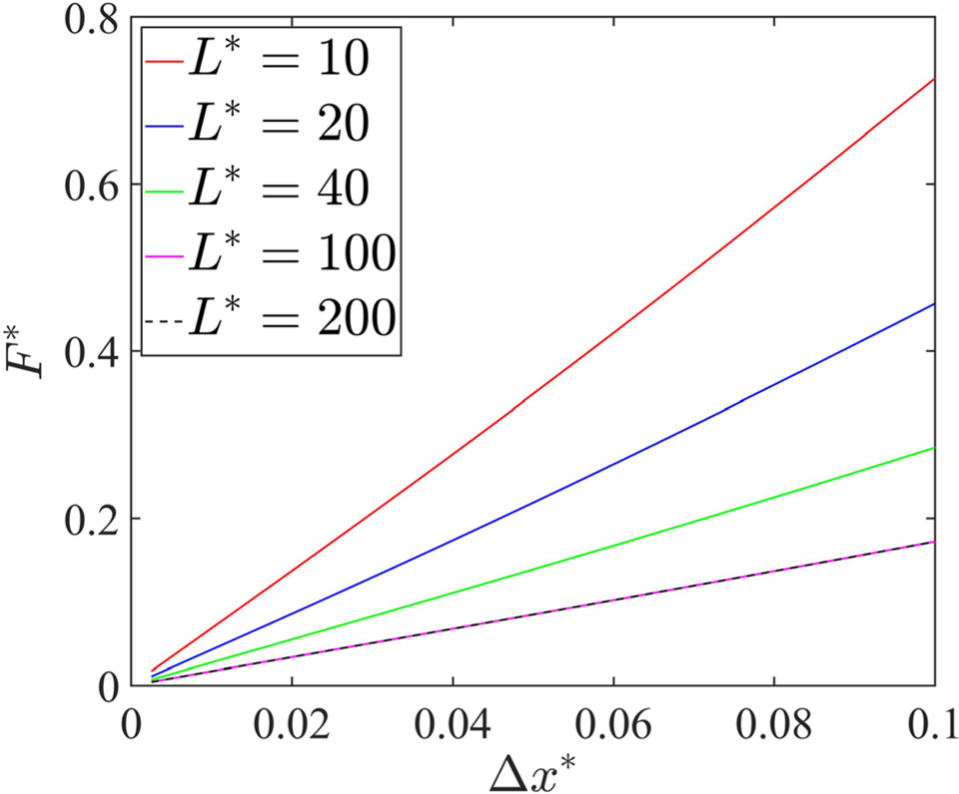
Force acting on the bead along *x*-direction $F^*$ versus displacement of the bead $\Delta x^*$ for the viscoelastic timescale $\tau _{\text{ve}}=1.0$ and the poroelastic timescale $\tau _{{\text{pe}}}=1.0$ obtained using Darcy flow model for various domain sizes $L^*=10$ (Red), $L^*=20$ (Blue), $L^*=40$ (Green), $L^*=100$ (Cyan), $L^*=200$ (Black)

To minimize the long-range influence of the outer boundaries of the computational domain, the domain size $L^*$ must be chosen significantly larger than the bead size. Figure 3 shows the force acting on the bead along *x*-direction ($F^*=\varvec{F}^*\cdot \hat{\varvec{x}}$) versus bead displacement ($\Delta x^*$) curves for several domain sizes under Darcy flow conditions with $\tau _{\text{ve}}=1.0$ and $\tau _{\text{pe}}=1.0$, indicating that the force-displacement curves are identical for domain sizes $L^*=100$ and $L^*=200$. We use $L^*=100$ throughout the remainder of the study.

## Results

In this section, we characterise the rheology of our model cytoplasm, investigating the effect of the governing parameters presented in Table 1. Similar to the experiments (Hu et al. 2017), we examine the characteristics of the component of the force acting on the bead, $F^*$, in the direction of bead movement. In addition to this, our computational simulations allow us to separately examine the contributions of the viscoelastic skeleton through the skeleton force $F^*_{{\text{sk}}}=\varvec{F}^*_{\text{sk}}\cdot \hat{\varvec{x}}$ and the pore fluid via the pore fluid force $F^*_{\text{pore}}=\varvec{F}_{\text{pore}}^*\cdot \hat{\varvec{x}}$.

We characterise the stiffness of the cytoplasm through the non-dimensional apparent Young’s modulus $E_A^*$ (Hu et al. 2017), which we compute as the average slope of the force per unit area $F^*$ versus displacement $\Delta x^*$ curve during the loading phase, excluding the first *M* timesteps corresponding to the initial $10\%$ of the bead displacement, given as

45 $$\begin{aligned} E_A^*=\frac{1}{N-M}\sum _{n=M}^{N-1} \frac{F^{*,n+1}-F^{*,n}}{\Delta x^{*,n+1}-\Delta x^{*,n}}, \end{aligned}$$

in which *N* is the total number of timesteps during the loading phase. The dimensional forms of the apparent Young’s modulus ($E_A$), and the forces *F*, $F_{\text{sk}}$, and $F_{\text{pore}}$ are easily obtained by multiplying their corresponding non-dimensional forms with the filament stretching stiffness *c*.

In Sect. 5.1, we first present results without the viscous effects of the pore fluid using the Darcy flow model (Eq. 36e) for various viscoelastic and poroelastic timescales $\tau _{\text{ve}}$ and $\tau _{\text{pe}}$. Subsequently, in Sect. 5.2, we generalise the predictions to incorporate the Brinkman flow model (Eq. 36f), investigating the additional role of the (non-dimensional) viscous timescale $\tau _{\text{vis}}$.

### Darcy flow

In this section, we begin by illustrating an overview of the model predictions in the form of a regime diagram, across the parameter space spanned by the viscoelastic timescale $\tau _{\text{ve}}$ and poroelastic timescale $\tau _{\text{pe}}$, plotting a colour contour of the apparent Young’s modulus, $E_A$. We identify seven baseline regimes corresponding to different values of $\tau _{\text{ve}}$ and $\tau _{\text{pe}}$. For each regime, we provide a detailed analysis of its characteristics, decomposing the rheological response into components from the viscoelastic skeleton and the pore fluid flow. Finally, we present an overview of the parameter space, quantifying the boundaries between the regimes.

#### Phase diagram

**Fig. 4:**
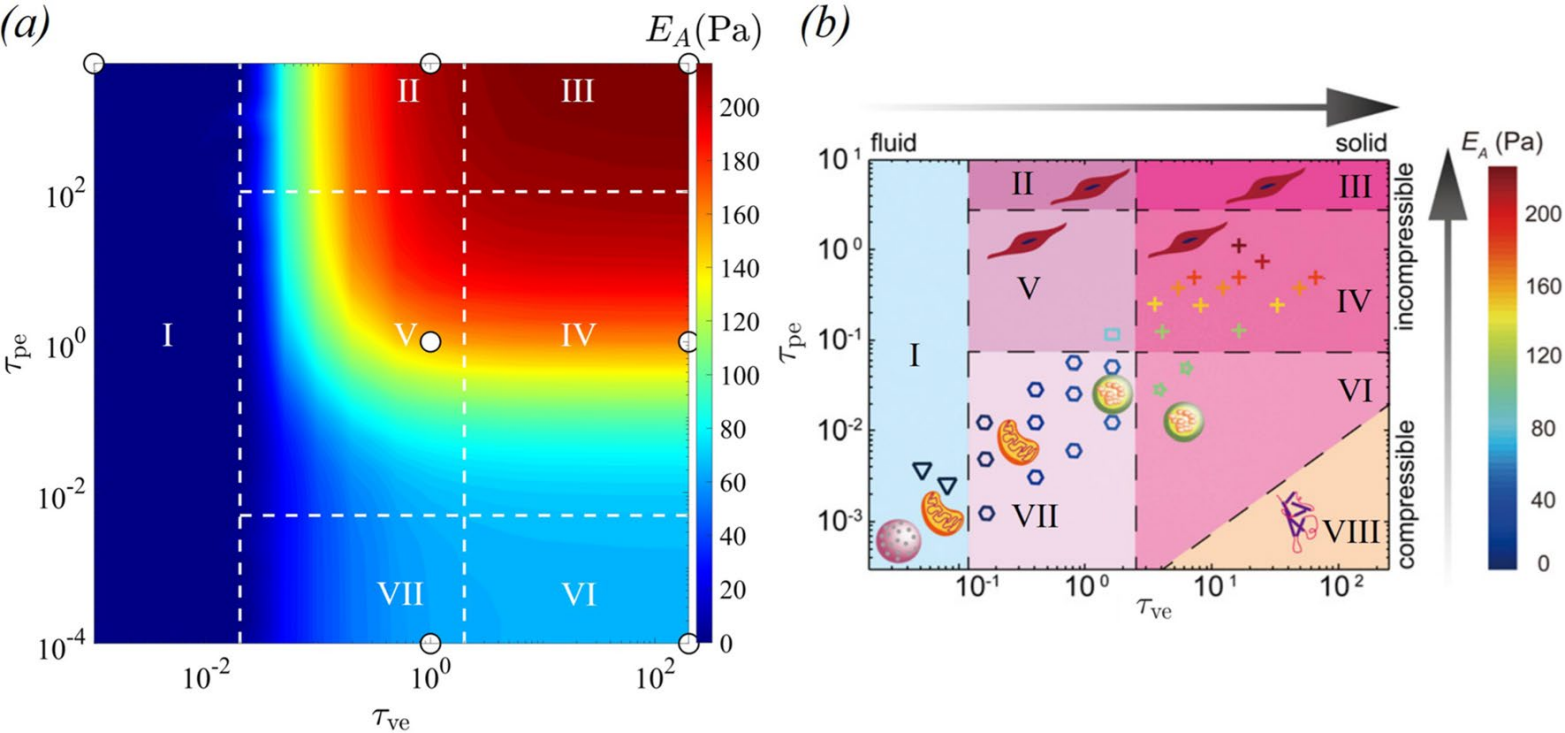
Contour plot of the apparent Young’s modulus $E_A$ as a function of the viscoelastic timescale $\tau _{\text{ve}}$ and the poroelastic timescale $\tau _{\text{pe}}$ obtained from: **a** our numerical simulations using Darcy flow, **b** optical tweezers experiments of Hu et al. (2017) (Modified from the figure presented by Hu et al. 2017). The regimes marked above represent **I** Viscous, **II** Viscoelastic, **III** Elastic, **IV** Poroelastic, **V** Poroviscoelastic, **VI** Compressible elastic, and **VII** Compressible viscoelastic. The white circles in (**a**) represent the baseline cases discussed in Sect. 5.1.2

To first provide an overview of the different rheological responses, in Fig. 4 we provide a contour plot of the apparent Young’s modulus $E_A$ as a function of the viscoelastic and poroelastic timescales. Across the parameter space, we identify seven different regimes which we label as I–VII and analyse each in detail in Sect. 5.1.2. We rationally identify boundaries between these regimes through analysis of the logarithmic gradient of the apparent Young’s modulus in Sect. 5.1.3; for clarity, these boundaries are marked in Fig. 4a.

Our seven regimes correspond qualitatively to regimes identified by the optical tweezers experiments of Hu et al. (2017) spanning the same parameter space (reproduced in Fig. 4b). Our colour contour plot exhibits strong qualitative agreement with the experimental plot in all regimes, except for regime VIII, where the poroelastic continuum approach does not contain the required ingredients to characterise a fibrous material.

Note that the results presented here are based on a fixed pre-stretch of $\alpha =1.012$. While varying pre-stretch values, the qualitative nature of the regime diagram remains consistent. The influence of pre-stretch on the apparent Young’s modulus across different regimes is detailed in Appendix 2.

#### Baseline cases

**Fig. 5:**
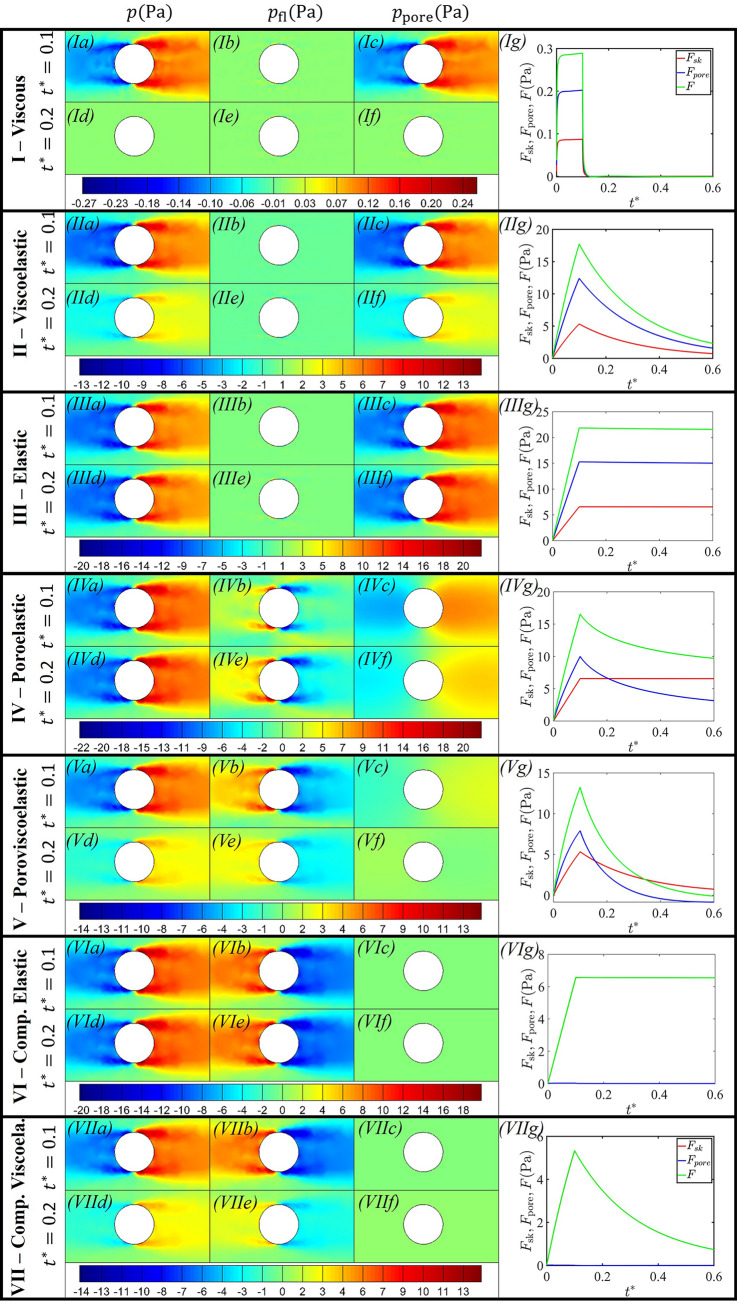
Fields of the skeleton pressure *p*, fluid pressure $p_{\text{fl}}$, and total pore pressure $p_{\text{pore}}$ at the end of loading $t^*=0.1$ and during relaxation $t^*=0.2$ along with the timewise variation of the skeleton force $F_{\text{sk}}$, the pore fluid $F_{\text{pore}}$, and the total force *F* in regime I (Viscous fluid) with $\tau _{\text{ve}}=10^{-3}$ and $\tau _{\text{pe}}=5\times 10^3$, regime II (Viscoelastic) with $\tau _{\text{ve}}=1.0$ and $\tau _{\text{pe}}=5\times 10^3$, regime III (Elastic) with $\tau _{\text{ve}}=2\times 10^2$ and $\tau _{\text{pe}}=5\times 10^3$, regime IV (Poroelastic) with $\tau _{\text{ve}}=2\times 10^2$ and $\tau _{\text{pe}}=1.0$, regime V (Poroviscoelastic) with $\tau _{\text{ve}}=1.0$ and $\tau _{\text{pe}}=1.0$, regime VI (Compressible Elastic) with $\tau _{\text{ve}}=2\times 10^2$ and $\tau _{\text{pe}}=10^{-4}$, and regime VII (Compressible Viscoelastic) with $\tau _{\text{ve}}=1.0$ and $\tau _{\text{pe}}=10^{-4}$

In order to assess the flow fields in the neighbourhood of the moving bead under the assumption of Darcy flow, Fig. 5 presents these seven baseline cases with a loading time of $t^*=0.1$. Similar to the experimental results, we analyse the variation of the total force acting on the bead per unit surface area *F* (Fig. 5) during the loading and relaxation periods. Additionally, our simulation results provide contour plots illustrating the variation of the skeleton pressure *p* (Fig. 5I–VII, a, d), pore fluid pressure due to change in pore volume $p_{\text{fl}}$ (Fig. 5I–VII, b, e), and the total pore pressure $p_{\text{pore}}$ (Fig. 5I–VII, c, f) near the wake and leading edge of the bead, along with the individual contributions to the total force from the viscoelastic skeleton $F_{\text{sk}}$ and the pore fluid $F_{\text{pore}}$ (Fig. 5I–VII, g). This comprehensive analysis provides detailed insight into the characteristics of the cytoplasm and the distinct influences of the viscoelastic skeleton and pore fluid on the rheology; such a decomposition is challenging to achieve through experimental results alone.

In regime I, for a short viscoelastic timescale compared to the loading timescale ($\tau _{\text{ve}}=10^{-3}$) and a poroelastic timescale much longer than the loading timescale ($\tau _{\text{pe}}=5\times 10^3$), Fig. 5Ia shows that as the bead is displaced, stretch of the skeleton in the wake drives a negative pressure, while compression of the skeleton near the leading edge of the bead drives positive pressure. Consequently, this pressure difference drives pore fluid into the wake of the moving bead. However, the magnitude of the fluid pressure $p_{\text{fl}}$ is considerably smaller than the skeleton pressure *p* (Fig. 5Ia, Ib), and the total pore pressure $p_{\text{pore}}$ is nearly identical to the solid pressure *p* (Fig. 5Ic). As a result, the skeleton force per unit area $F_{\text{sk}}$, the pore fluid force unit area $F_{\text{pore}}$, and the total force per unit area *F* are almost identical (Fig. 5Ig). During the loading period, *F* initially increases rapidly and then remains approximately independent of the bead’s displacement (Fig. 5Ig), similar to the experimental observations of viscous behaviour reported by Hu et al. (2017). The initial overshoot in force is a direct consequence of the simulation/experimental setup, which commences with zero velocity and rapidly transitions to a constant value. After the initial phase, the force acting on the bead is independent of the displacement of the bead or the extent of the stretch of the filaments. Furthermore, during the relaxation phase, the pressures and the corresponding forces quickly relax to zero with a very short relaxation time. These characteristics are typical of a viscous fluid. We also note that characterising the material by an apparent Young’s modulus (based on the gradient of the force-displacement curve) is an ineffective measure in this regime.

In regime II, for a viscoelastic timescale comparable to the loading timescale ($\tau _{\text{ve}}=1.0$) and a poroelastic timescale much longer than the loading timescale ($\tau _{\text{pe}}=5\times 10^3$), Fig. 5IIa–IIb shows that the fluid pressure $p_{\text{fl}}$ is negligible compared to the skeleton pressure *p*, attributed to minimal fluid movement caused by the extremely low permeability of the skeleton. Unlike the viscous fluid behaviour in regime I, the bead force increases almost linearly with the displacement during the loading period (Fig. 5IIg). During relaxation, the pressure *p* and the pore pressure $p_{\text{pore}}$ gradually relaxes (Fig. 5IId, IIf) with a gradual decline in the forces, eventually settling at a constant value (approximately zero) over a time span of approximately ${\mathcal {O}}(1)$ (Fig. 5IIg). The nearly linear increase in force during the loading phase and its exponential decrease during relaxation correspond qualitatively with the experimental observations (Hu et al. 2017). Since the fluid pressure $p_{\text{fl}}$ is negligible (Fig. 5IIb, IIe), relaxation is solely due to the viscoelastic relaxation of the skeleton. Consequently, the cytoplasm essentially behaves as a viscoelastic material in this regime with an ${\mathcal {O}}(1)$ viscoelastic relaxation time.

In regime III, for viscoelastic and poroelastic timescales much longer than the loading timescale ($\tau _{\text{ve}}=2\times 10^2$ and $\tau _{\text{pe}}=5\times 10^3$), similar to Fig. 5Ib and IIb, the fluid pressure $p_{\text{fl}}$ is negligible (Fig. 5IIIb). The total force increases almost perfectly linearly with increasing displacement (Fig. 5IIIg). During the relaxation phase, the skeleton pressure *p*, the pore pressure $p_{\text{pore}}$, and the components of forces remain almost unchanged over the interval considered (Fig. 5IIIg). The time-independence of the relaxation curve shows that the cytoplasm essentially behaves as an elastic material.

In regime IV, for a viscoelastic timescale much longer than the loading timescale ($\tau _{\text{ve}}=2\times 10^2$) and a poroelastic timescale comparable to the loading timescale ($\tau _{\text{pe}}=1$), the permeability of the porous medium is sufficiently large to facilitate considerable pore fluid movement. So, the fluid pressure $p_{\text{fl}}$ (Fig. 5IVb) is comparable to that of the skeleton pressure *p*. As a result, the total pore pressure $p_{\text{pore}}$ includes the combined effects of the elastic behaviour of the skeleton and the pore fluid movement (Fig. 5IVc). Since the skeleton approximately behaves as an elastic material, during relaxation, the skeleton pressure *p* is consistently larger near the leading edge of the bead and smaller in the wake. So, the fluid continues to move into the wake until the material is fully relaxed when the total pore pressure $p_{\text{pore}}$ becomes equal on both sides. Consequently, the difference in pore fluid pressure $p_{\text{fl}}$ between the leading and trailing surfaces transiently becomes more pronounced during the relaxation (compare $t^*=0.2$ to $t^*=0.1$); note that *p* and $p_{\text{fl}}$ have opposite signs, resulting in a decrease in the total pore pressure $p_{\text{pore}}$ during relaxation (e.g. $t^*=0.2$, Fig. 5IVf). In the relaxation phase, the skeleton force $F_{\text{sk}}$ is almost uniform (Fig. 5IVg), whereas $F_{\text{pore}}$ decreases gradually and asymptotes to a constant value over an ${\mathcal {O}}(1)$ time interval. So, the gradual decrease in the total force *F* during the relaxation (Fig. 5IVg) is almost entirely due to the movement of the pore fluid, indicating that the cytoplasm essentially behaves as a poroelastic material with an ${\mathcal {O}}(1)$ relaxation time. Note that in the poroelastic regime, the pore fluid flow between the pores causes localized changes in pore volume and local regions of material compression.

In regime V, for viscoelastic and poroelastic timescales comparable to the loading timescale ($\tau _{\text{ve}}=1$ and $\tau _{\text{pe}}=1$), the skeleton behaves as a viscoelastic material (Fig. 5Va, Vg). However, unlike the viscoelastic regime (Fig. 5IIb, IIe), the fluid pressure $p_{\text{fl}}$ is comparable to the skeleton pressure (Fig. 5Vb, Ve). Similar to the poroelastic regime (Fig. 5IV), the pressure gradient drives the fluid into the wake of the moving bead, resulting in larger $p_{\text{fl}}$ in the wake and smaller $p_{\text{fl}}$ near the leading edge of the bead (Fig. 5Vb). During relaxation, the skeleton pressure *p* (Fig. 5Va), and hence the skeleton force $F_{\text{sk}}$ (Fig. 5Vg), gradually relaxes due to viscoelastic effects. At the same time, the pore fluid flow also reaches a steady state (Fig. 5Vb, Ve). However, unlike regime IV (poroelastic regime,), where the fluid pressure $p_{\text{fl}}$ increases uniformly during relaxation, here $p_{\text{fl}}$ decreases uniformly during relaxation, as a result of the reduction in the skeleton pressure *p*, which drives the pore fluid flow. With the combined relaxation due to viscoelasticity and poroelasticity, the pore pressure $p_{\text{pore}}$ decreases more rapidly than either poroelastic or viscoelastic materials alone (with the same parameter values), resulting in almost zero pore pressure by $t^*=0.2$ (Fig. 5Vf). Similarly, the total force also asymptotes to a constant value more quickly than due to viscoelasticity alone (Fig. 5Vg). Since both the pore fluid and viscoelastic relaxation play significant roles, the cytoplasm essentially behaves as a poroviscoelastic material.

In regime VI, for a viscoelastic timescale much longer than the loading timescale ($\tau _{\text{ve}}=2\times 10^2$) and a poroelastic timescale much shorter than the loading timescale ($\tau _{\text{pe}}=10^{-4}$), the relaxation of the skeleton pressure (Fig. 5VIa, VId) is similar to that of an elastic material (Fig. 5VIg). However, the significantly large permeability of the porous medium results in substantial pore fluid movement and the pore fluid pressure $p_{\text{fl}}$ is comparable to the skeleton pressure *p* (Fig. 5VIb). Due to the larger permeability, the pore fluid moves quickly to the wake of the bead. Thus, the skeleton pressure *p* and the fluid pressure $p_{\text{fl}}$ balance, leading to almost zero pore pressure $p_{\text{pore}}$ (Fig. 5VIc, VIf). The total force *F* is dominated by the skeleton pressure *p* with a very long relaxation time (Fig. 5VIg). Consequently, the cytoplasm behaves as a compressible elastic material (compressibility due to the movement of pore fluid).

In regime VII, for a viscoelastic timescale comparable to the loading timescale ($\tau _{\text{ve}}=1.0$) and a poroelastic timescale much shorter than the loading timescale ($\tau _{\text{pe}}=10^{-4}$), the skeleton pressure (Fig. 5VIIa, VIId) and the skeleton force (Fig. 5VIIg) are almost identical to that of a viscoelastic material (Fig. 5II). Similar to regime VI (Fig. 5VI), the pore fluid movement is much faster than the viscoelastic relaxation, resulting in large volume changes, and so the pore pressure rapidly reaches a state of balance, where the pore pressure $p_{\text{pore}}$ is negligible (Fig. 5VIIc, VIIf). Thus, the relaxation of the total force per unit area *F* is dominated by the viscoelastic response of the skeleton, and so we characterise the overall behaviour in this regime as compressible viscoelastic.

#### Overview of parameter space

We note that our seven representative cases agree qualitatively with the experimental parameter space presented by Hu et al. (2017). Given the baseline cases discussed in Sect. 5.1, we now attempt to overview the parameter space spanned by $\tau _{\text{ve}}$ and $\tau _{\text{pe}}$ and divide up the phase space in a more systematic manner.

To better quantify transitions between regimes of cytoplasmic behaviour, we compute the logarithmic gradients of the non-dimensional apparent Young’s modulus $E_A^*$ with respect to $\tau _{\text{ve}}$ and $\tau _{\text{pe}}$, given as

46 $$\begin{aligned} gE_{A,\text{ve}} = \frac{\text{d}(E_A^*)}{\text{d}\log (\tau _{\text{ve}})},\quad gE_{A,{\text{pe}}} = \frac{\text{d}(E_A^*)}{\text{d}\log (\tau _{\text{pe}})}. \end{aligned}$$

**Fig. 6:**
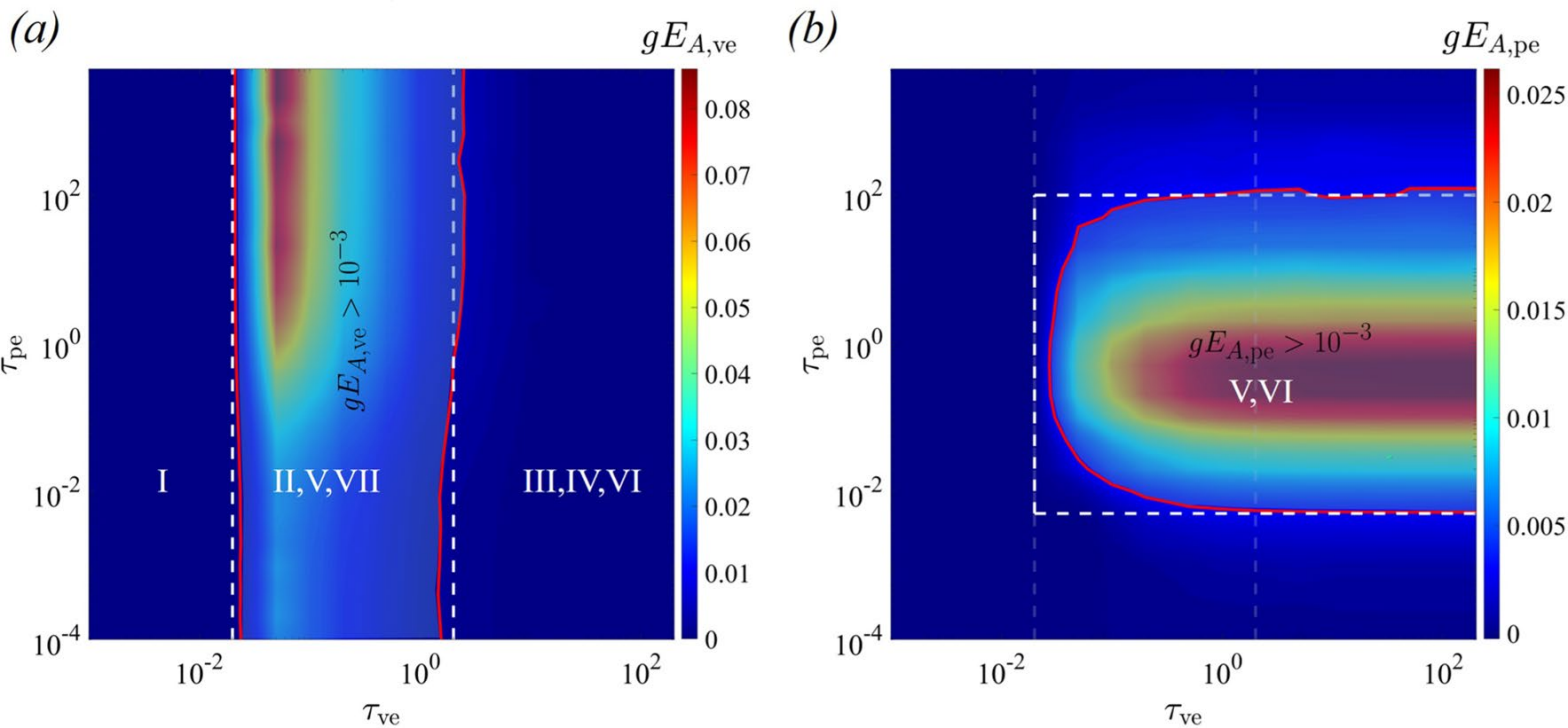
**a** Contour plot of the logarithmic gradients of the apparent Young’s modulus (a) $gE_{A,\text{ve}}$ and **b** $gE_{A,{\text{pe}}}$ as a function of the viscoelastic timescale $\tau _{\text{ve}}$ and poroelastic timescale $\tau _{\text{pe}}$. Red lines in (**a**) and (**b**) correspond to $gE_{A,\text{ve}}=10^{-3}$ and $gE_{A,{\text{pe}}}=10^{-3}$, respectively

**Fig. 7:**
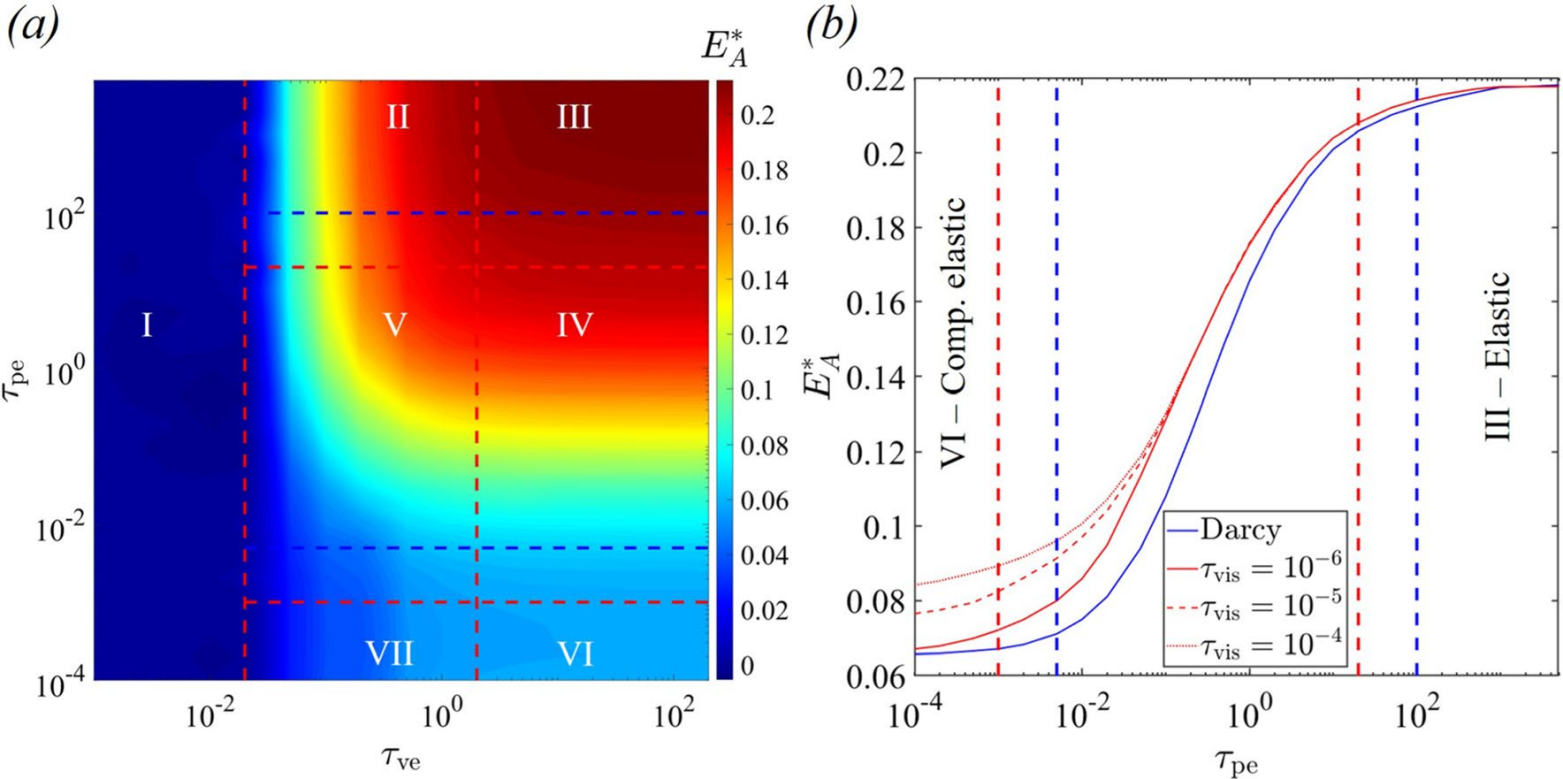
**a** Contour plot of the non-dimensional apparent Young’s modulus $E_A^*$ as a function of the viscoelastic timescale $\tau _{\text{ve}}$ and the poroelastic timescale $\tau _{\text{pe}}$ at viscous timescale $\tau _{\text{vis}}=10^{-6}$ and **b** variation of $E_A^*$ with $\tau _{\text{pe}}$ at $\tau _{\text{ve}}=200$ obtained using Darcy flow model and Brinkman flow model with various viscous timescales. In both figures, the red (blue) dotted straight lines show the boundaries of regimes for the Brinkman (Darcy) flow models

In the viscous regime, the apparent Young’s modulus $E_A^*$ is nearly zero and is unaffected by the viscoelastic timescale $\tau _{\text{ve}}$ and the poroelastic timescale $\tau _{\text{pe}}$ (Fig. 4a). Consequently, both $gE_{A,\text{ve}}$ and $gE_{A,{\text{pe}}}$ approach values close to zero. We characterise this regime for cases where both $gE_{A,\text{ve}}$ and $gE_{A,{\text{pe}}}$ are less than $10^{-3}$. The viscoelastic effect is significant within the cytoplasm if $E_A^*$ exhibits dependency on the viscoelastic timescale $\tau _{\text{ve}}$, while the poroelastic effect is present if $E_A^*$ shows dependence on the poroelastic timescale $\tau _{\text{ve}}$. Thus, we characterise the viscoelastic effects by $gE_{A,\text{ve}}>10^{-3}$ and poroelastic effects by $gE_{A,{\text{pe}}}>10^{-3}$. To demarcate the transition between the different regimes, we plot line contour corresponding to $gE_{A,\text{ve}}=10^{-3}$ (Fig. 6a) and $gE_{A,{\text{pe}}}=10^{-3}$ (Fig. 6b). Figure 6a shows that the viscoelastic effects are restricted in the region between the two vertical lines, whereas Fig. 6b shows that the poroelastic effects are significant in the region within the convex area of the red curve. For simplicity, we approximate the contours using horizontal and vertical lines, shown by white dashed lines (Fig. 6). These lines mark the boundaries of transition between the seven regimes. Note that these boundaries are used in our parameter space plot in Fig. 4a; the same approach is used to demarcate the regimes in Fig. 7a.

**Fig. 9:**
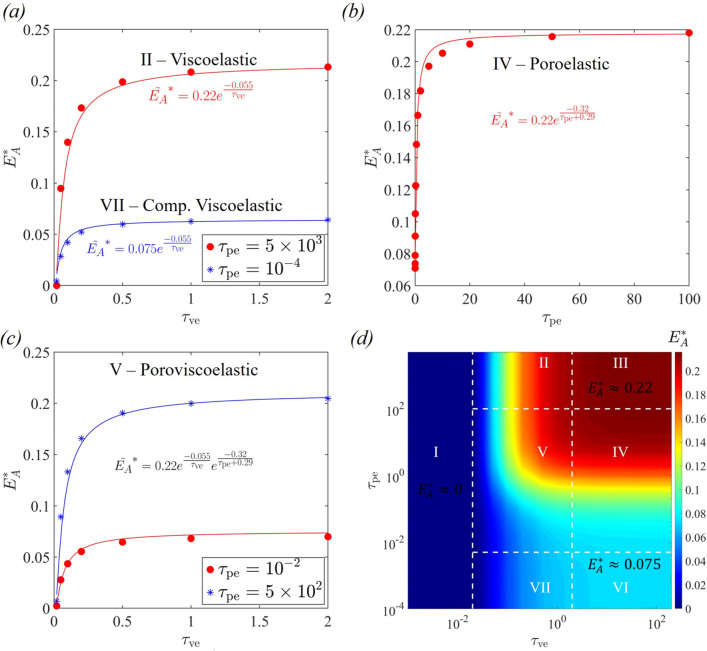
Variation of the non-dimensional apparent Young’s modulus $E_A^*$ obtained from the numerical simulations using Darcy flow model and a nonlinear curve fitting in **a** Viscoelastic regime at $\tau _{\text{pe}}=5\times 10^{3}$ and compressible viscoelastic regimes at $\tau _{\text{pe}}=10^{-4}$, **b** Poroelastic regime at $\tau _{\text{ve}}=200$, and **c** Poroviscoelastic regime at $\tau _{\text{pe}}=10^{-2}$ and $\tau _{\text{pe}}=500$, and **d** the contour plot of $E_A^*$ as a function of $\tau _{\text{ve}}$ and $\tau _{\text{pe}}$ obtained from the nonlinear curve fitting

Through a nonlinear curve fitting, we have established an exponential relationship between $E_A^*$ and $\tau _{\text{ve}}$ and $\tau _{\text{pe}}$ in regimes I–VII (“Appendix 3”). Figure 9 demonstrates that the regime map obtained using our model aligns remarkably well with the simulation results.

### Brinkman flow

We now extend our analysis to consider the role of viscous flow in the pore fluid by assuming Brinkman flow in the pores. To this end, Fig. 7a shows a contour plot of the apparent Young’s modulus $E_A^*$ with a finite viscous timescale $\tau _{\text{vis}}=10^{-6}$. When compared to Darcy flow (Fig. 4a), we observed that Brinkman flow drives a shift in the system, resulting in a reduction of the critical values of the poroelastic timescale $\tau _{\text{pe}}$ at which transition from regimes III to IV and IV to VI occurs, i.e. the transition between the regimes are shifted to shorter poroelastic timescales.

When the poroelastic timescale $\tau _{\text{pe}}$ is sufficiently long, the Darcy and Brinkman flow models closely align, yielding an identical prediction for the apparent Young’s modulus $E_A^*$ due to negligible pore fluid flow and viscous effects. At moderate values of $\tau _{\text{pe}}$, Brinkman flow yields larger values of $E_A^*$ as compared to the Darcy flow (Fig. 7b) because viscous effects inhibit the movement of the pore fluid, and the cytoplasm effectively becomes increasingly incompressible. As a result, Brinkman flow yields a slightly larger pore pressure difference across the bead surface. Due to increased incompressibility induced by the viscous effects, the critical value of the poroelastic timescale $\tau _{\text{pe}}$, marking the transition from regimes III to IV (elastic to poroelastic), is reduced for Brinkman flow compared to Darcy flow. Similarly, the critical value of the poroelastic timescale $\tau _{\text{pe}}$ for the transition from IV to VI (poroelastic to compressible elastic) is also reduced for Brinkman flow compared to Darcy flow. Considering the limitations of our model, especially its two-dimensional nature, it remains uncertain at this stage if the inclusion of Brinkman flow will result in a significant enhancement in quantitative agreement with the experimental results.

To further quantify the role of the pore fluid viscosity, Fig. 7b plots the apparent Young’s modulus $E_A^*$ as a function of the poroelastic timescale $\tau _{\text{pe}}$ for various values of the viscous timescale $\tau _{\text{vis}}$ (for a long viscoelastic timescale $\tau _{\text{ve}}=200$). It is evident that the viscous effects make negligible difference to the behaviour in the incompressible regime, but becomes increasingly important as the material becomes more compressible. In the compressible regime, a viscous timescale $\tau _{\text{vis}}=10^{-6}$ shows a substantial difference to Darcy flow, while for longer $\tau _{\text{vis}}$, the apparent Young’s modulus $E_A^*$ increases. Consequently, spatially non-uniform viscosity within the cytoplasm could lead to a spatial variation in the cytoplasm stiffness.

It is important to highlight that the viscosity of the pore fluid has an impact primarily on the critical values of the poroelastic timescales for transition. However, we note that the critical values of the viscoelastic timescales governing transitions between different regimes remain unaffected by the viscous effects because, for the parameters considered here, the viscoelastic relaxation of the skeleton network dominates the pore fluid viscous effects. In summary, the viscous effects of the pore fluid have (perhaps surprisingly) little effect on the viscoelastic response but make a significant difference to the poroelastic response.

## Conclusions

In this study, we have constructed a novel mathematical model for characterizing the rheology of the living cell cytoplasm; this model incorporates the interaction between the viscoelastic cytoskeletal network and cytosol flow through the filamentous mesh. In particular, our approach integrated a continuum hyperelastic model for the cytoskeletal filament network (based on rational upscaling of a discrete network model, Köry et al. 2024) with a Maxwell-type viscoelastic model (Unterberger et al. 2013) and a poroelastic model for the interaction between the cytoskeleton and the cytosol fluid (Moeendarbary et al. 2013). We constructed a numerical method to solve this model, employing a finite element method based on stabilized first-order elements.

This model was used to mimic optical tweezers experiments to characterise cell rheology (Hu et al. 2017), calculating the force required to move a circular bead through the medium as a function of its displacement. In particular, we characterised the response in terms of the timescales of viscoelastic relaxation and poroelastic diffusion, respectively, relative to the timescale of motion of the bead. Through numerical simulations, we demonstrated how the model successfully captured the rheology of the cell cytoplasm, demonstrating seven different responses including purely viscous (Fig. 5I), purely viscoelastic (both compressible and incompressible, Fig. 5II, VII), purely elastic (both compressible and incompressible, Fig. 5III, VI), poroelastic (Fig. 5IV) and poroviscoelastic (Fig. 5V); the computed phase boundaries between these responses (in the parameter space spanned by the poroelastic and viscoelastic timescales) demonstrates excellent qualitative agreement with the experiments (Hu et al. 2017).

Having successfully reproduced the rheological behaviour evident in the optical tweezers experiments, we further extended our modelling approach to consider the additional role of the pore fluid viscosity by including a Brinkman term in the poroelastic component of the model (Eq. 12), constructing an additional dimensionless timescale. Using numerical simulations, we demonstrated that increasing the pore fluid viscosity shifts the numerically computed phase boundaries towards lower poroelastic timescales (but makes almost no difference to the phase boundaries set by the viscoelastic timescale), rendering the overall rheological behaviour of the cell increasingly incompressible.

### Limitations and future work

Our model incorporated most of the structural components of the cytoplasm. However, for simplicity, we only considered a single family of pre-stretched cytoskeletal filaments, and we avoided additional complexities by excluding the effects of structural components such as organelles. Thus, unlike tensegrity models, where individual elements can exhibit stretching or compression (Bansod et al. 2018; Stamenovic and Coughlin 2000), we represent pre-stretch with a single family of tensioned filaments. Our modelling framework has the potential to expand to include two or more cytoskeletal components (such as actin stress filaments, and microtubules), each exhibiting varying degrees of pre-stretch or pre-compression.

For the fluid component of the cytoplasm, we assumed that fluid flow is solely driven by pore pressure gradients, neglecting fluid movement caused by internal cellular processes, such as those powered by molecular motors. Although molecular motor-driven forces can induce vortex-like rotational flows in larger cells (Suzuki et al. 2017), their impact is generally minimal in smaller cells (Mogilner and Manhart 2018), validating our assumption for smaller cells. Furthermore, we simplified the model by computing the pore pressure under the assumption of a spherical pore instead of the actual cytoskeleton geometry. While this model may not precisely replicate fluid flow patterns within the cytoplasm, simplified pore fluid models are sufficient to capture poroelastic behaviour (Moeendarbary et al. 2013). Additionally, we assumed uniform viscosity throughout the cytoplasm, disregarding variations caused by long-range ordering effects in the cytosol. In future work, we aim to incorporate the effect of molecular motor-driven fluid flow and develop a rational model for the porous flow based on the actual cytoskeletal geometry with variable viscosity in the cytoplasm.

It is worth noting that the viscoelastic relaxation behaviour of the cytoplasm may exhibit distinct time scales, including both slow and fast relaxation, ultimately resulting in power-law relaxation (Hu et al. 2019). So, a single viscoelastic time scale proposed in this study cannot fully account for this power-law behaviour. Future research will investigate the influence of multiple viscoelastic time scales. Additionally, the model introduced here primarily addresses small skeletal deformations and does not account for filament breakage. However, filament breakage becomes a critical factor for large deformations and will be investigated in future work.

The model presented in this study is a two-dimensional planar representation of the cytoplasm. Moreover, we assume the computational domain to be rectangular and that the bead is positioned well away from the boundary. Given the two-dimensional nature of our model and the aforementioned limitations, our study is restricted to a qualitative comparison with experimental findings. A quantitative assessment will be pursued in subsequent work, using a more advanced three-dimensional model with additional structural components.

## Figures and Tables

**Fig. 8:**
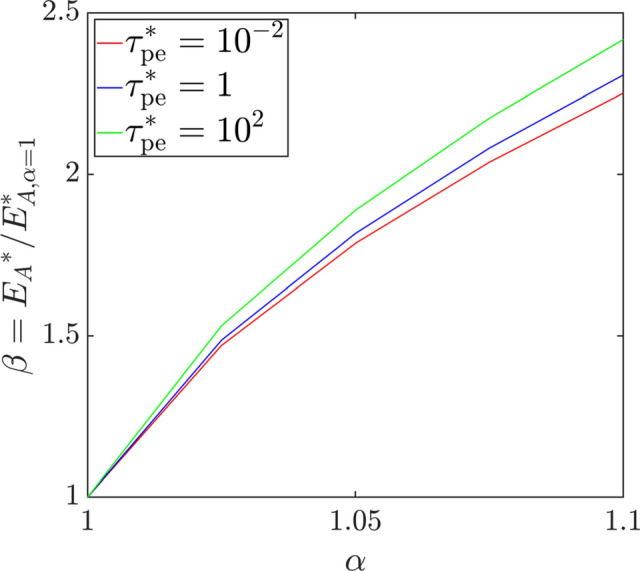
Variation of the ratio of the apparent Young’s modulus $E_A^*$ with pre-stretch to without pre-stretch $E_{A,\alpha =1}^*$ for various poroelastic timescales at a constant viscoelastic timescale $\tau _{\text{ve}}=10^2$

## Appendix 1: Pore fluid pressure

We assume that each pore is a spherical shell characterized by an inner radius *R* and thickness *H* in the reference configuration, with the deformation of the pores limited to the radial direction. Additionally, we assume that the spherical shell is an incompressible neo-Hookean material, with Young’s modulus *E*. Consequently, the shear modulus of this incompressible neo-Hookean material is $G = E/3$.

For the radial deformation of the spherical shell with deformed inner radius *r*, and inflation stretch $\lambda =r/R$, the incompressibility condition gives the following principal stretches (Anssari-Benam et al. 2021)

47 $$\begin{aligned} \lambda _{rr} = \lambda ^{-2}, \quad \lambda _{\theta \theta }=\lambda _{\phi \phi } = \lambda , \end{aligned}$$

and the corresponding Cauchy stress tensor in the spherical coordinate system, given as

48 $$\begin{aligned} \sigma _{\text{shell}} = G\text{diag}\left( \lambda ^{-4},\lambda ^2,\lambda ^2\right) - p_{\text{shell}}\varvec{I} \end{aligned}$$

in which $p_{\text{shell}}$ is the hydrostatic pressure. Using thin shell assumption, the balance of resistance force and the boundary force due to the fluid pressure $p_{\text{fl}}$ at the inner surface gives (Anssari-Benam et al. 2021)

49 $$\begin{aligned} \frac{p_{\text{fl}}R}{2H}\lambda ^3=\sigma _{\theta \theta }=\sigma _{\phi \phi } \end{aligned}$$

Substituting for the tangential stresses from Eq. 48, the above equation gives

50 $$\begin{aligned} p_{\text{fl}}=\frac{2GH}{R}\left( \lambda ^{-1}-\lambda ^{-7}\right) \end{aligned}$$

The ratio of the volume of the pore fluid in the current configuration to the reference configuration is given as

51 $$\begin{aligned} 1+\Delta V_{\text{f}}=\lambda ^3 \end{aligned}$$

Substituting $\lambda$ in terms of $\Delta V_{\text{f}}$ in Eq. (50), the pore fluid pressure $p_{\text{fl}}$ is obtained in terms of the volume change ratio $\Delta V_{\text{f}}$, given as

52 $$\begin{aligned} p_{\text{fl}}=\frac{2GH}{R}\left[ \left( 1+\Delta V_{\text{f}}\right) ^{-1/3}-\left( 1+\Delta V_{\text{f}}\right) ^{-7/3}\right] . \end{aligned}$$

The corresponding free energy density of the pore fluid written as a function of the pore volume change $\Delta V_{\text{f}}$ is obtained by integrating $p_{\text{fl}}$ with respect to $\Delta V_{\text{f}}$, given as

53 $$\begin{aligned} \Psi _{\text{fl}}(\Delta V_{\text{f}})=\frac{2GH}{R}\left[ \frac{3}{2}\left( 1+\Delta V_{\text{f}}\right) ^{2/3}+\frac{3}{4}\left( 1+\Delta V_{\text{f}}\right) ^{-4/3}\right] . \end{aligned}$$

To close the model, we must link the Young’s modulus *E* of the hyperelastic shell to the elastic properties of the skeleton. As a first approximation we set $E=c$, so that $G=c/3$, from which the free energy (Eq. 53) then follows

54 $$\begin{aligned} \Psi _{\text{fl}}(\Delta V_{\text{f}})=\frac{2cH}{3R}\left[ \frac{3}{2}\left( 1+\Delta V_{\text{f}}\right) ^{2/3}+\frac{3}{4}\left( 1+\Delta V_{\text{f}}\right) ^{-4/3}\right] . \end{aligned}$$

## Appendix 2: Effect of pre-stretch

To analyse the impact of pre-stretch on the apparent Young’s modulus $E_A^*$, we perform numerical simulations across a wide range of the parameter $\alpha$. Figure 8 shows the variation in the ratio of the apparent Young’s modulus computed with and without pre-stretch, $\beta \equiv E_A^*/E^*_{A,\alpha = 1}$, as a function of $\alpha$ for different values of the poroelastic timescale $\tau _{\text{pe}}$ at a constant viscoelastic timescale $\tau _{\text{ve}}=10^2$. As expected, an increase in pre-stretch leads to a stiffer cytoplasm, resulting in higher values of the apparent Young’s modulus ($\beta >1$). Notably, the rate of increase in $\beta$ with pre-stretch is more pronounced in the incompressible elastic regime (larger values of $\tau _{\text{pe}}$) compared to the compressible regime. Moreover, the network stiffness shows a slightly sublinear increase with pre-stretch across all regimes, which is consistent with the results in Köry et al. (2023).

## Appendix 3: Nonlinear curve fitting

A nonlinear curve fitting is performed to explore the relationship between the apparent Young’s modulus $E_A^*$ and the viscoelastic timescale $\tau _{\text{ve}}$ as well as the poroelastic timescale $\tau _{\text{pe}}$. In the viscoelastic (regime II) and compressible viscoelastic (regime VII) regimes, $E_A^*$ exhibits an exponential relationship with the viscoelastic time scale $\tau _{\text{ve}}$ (Fig. 9a). This is similar to the exponential relationship between the stress term and the viscoelastic timescale (Eq. 40). In the poroelastic regime (regime IV), a similar exponential relationship is obtained between the apparent Young’s modulus $E_A^*$ and the poroelastic timescale $\tau _{\text{pe}}$ (Fig. 9b). In the poroviscoelastic regime (regime V), $E_A^*$ exhibits an exponential dependence on both the timescales. Figure 9d shows a contour plot of the apparent Young’s modulus $E_A^*$ obtained through the nonlinear curve fitting, showing excellent agreement with the simulation results (Fig. 4a).
